# Early long-term low-dosage colchicine and major adverse cardiovascular events in patients with acute myocardial infarction: a systematic review and meta-analysis

**DOI:** 10.3389/fcvm.2023.1194605

**Published:** 2023-08-07

**Authors:** Yifang Zhou, Yidan Liu, Ruixiang Zeng, Wenjie Qiu, Yunhong Zhao, Yuanshen Zhou

**Affiliations:** ^1^ The Second Clinical College of Guangzhou University of Chinese Medicine, Guangzhou, China; ^2^ Department of Critical Care Medicine, Guangdong Provincial Hospital of Chinese Medicine, Guangzhou, China; ^3^ Department of Critical Care Medicine, Nanxiong City Hospital of Chinese Medicine, Shaoguan, China

**Keywords:** colchicine, myocardial infarction, major adverse cardiovascular events, CRP, inflammation

## Abstract

**Background:**

Current evidence on the efficacy and safety of colchicine after acute myocardial infarction (AMI) remains controversial. This study aims to clarify early low-dose long-term colchicine's exact efficacy and safety in AMI patients via more studies.

**Methods:**

We searched PubMed, Web of Science, Embase, and Cochrane Library databases for randomized controlled trials assessing the efficacy of colchicine on major adverse cardiovascular events (MACE) in recent AMI patients from inception to January 29, 2023, without any restriction. Additionally, we conducted subgroup analyses to assess the impact of early (≤3 days) long-term (≥1 year) low-dosage (0.5 mg/d) colchicine. Summary estimates were computed using Mantel-Haenszel and reported as risk ratios (RRs) or standard mean differences (SMDs), mean differences (MDs) with 95% confidence intervals (CIs). Sensitivity analyses were performed to explore the potential sources of heterogeneity. Review Manager software was used for the meta-analysis.

**Results:**

Eight studies identified from 564 screened records were analyzed, with 5,872 patients after AMI. The length of follow-up varied from five days to 22.7 months, and 0.5–1.0 mg colchicine was administered daily. In summary, compared to the control group, colchicine reduced the occurrence of MACE (RR, 0.56; 95% CI, 0.48–0.67) with 2.99-fold gastrointestinal adverse events in patients with recent AMI. Moreover, the relation referred to a gradual decrease in the occurrence of MACE with a longer follow-up duration (≥1 year) and lower dosage (0.5 mg/d) without leading more gastrointestinal adverse events. Colchicine decreased the follow-up levels of C-reactive protein (CRP) (MD −0.66, 95% CI, −0.98– −0.35) and neutrophils (SMD −0.22, 95% CI, −0.39– −0.55) when the follow-up period was 30 days.

**Conclusion:**

Early long-term low-dose colchicine decreases the risk of MACE via anti-inflammation without leading more gastrointestinal adverse events in patients with AMI.

## 1. Introduction

Even with optimal medical therapy, post-acute myocardial infarction (AMI) patients continue to face a high risk of mortality and morbidity (1, 2). Hence, optimizing the current therapeutic strategies to improve cardiovascular outcomes after AMI is necessary and urgent.

Nearly all AMIs are triggered by thrombi associated with atherosclerosis (3). A critical contributor to atherosclerotic plaque progression and instability is inflammation (4), for example, the involvement of NLRP3 inflammasome (4). Additionally, the neutrophil-to-lymphocyte ratio (NLR) is positively correlated with C-reactive protein (CRP) levels and is associated with in-hospital major adverse cardiovascular events (MACEs) (5). Furthermore, despite timely percutaneous coronary intervention (PCI), reperfussion injury also causes additional damage and inflammation (6, 7). Therefore, implementing the anti-inflammatory treatment makes sense.

Colchicine and canakinumab have been discovered to potentially reduce the incidence of MACEs in AMI settings (8). However, canakinumab is linked to a higher incidence of fatal infections (9). There is growing evidence that colchicine reduces the incidence of MACE in the secondary prevention of cardiovascular (CV) events (10, 11). Consequently, colchicine has gathered increasing interest as a tolerable and affordable anti-inflammatory agent.

Colchicine may improve cardiovascular outcomes by suppressing activation of the NLRP3 inflammasome (12, 13). Animal experiments demonstrated that inhibition and disruption of major components of NLRP3 decline infarct size after ischemia-reperfusion (I/R), improve cardiac remodeling and fibrosis after AMI, and enhance cardiac contractile function (4).

Firstly, it remains controversial whether colchicine reduces the risk of MACE in patients with AMI (14–16). Secondly, the study population for meta-analyses of Diaz-Arocutipa et al. (15) and Mendoza et al. (17) included patients with unstable angina (UA), and UA patients were evenly and randomly distributed between the colchicine and control groups cannot be assured. Further, the inflammation levels were substantially higher in patients with AMI than UA (18). It may introduce biases. Thirdly, we included two more randomized controlled trials (RCTs) with 558 participants compared with previous analyses. Moreover, we excluded three RCTs, being included in the analysis of previous studies, of patients with acute coronary syndromes (ACS) from the study, as follows Akrami et al. 2021 (19) (AMI: UA = 65.5%: 34.5%), Raju et al. 2012 (20) (AMI: UA: stroke = 77.5%: 13.75%: 8.75%) and Tong et al. 2020 (21) (AMI: UA = 96.7%: 3.3%). Finally, we aimed to clarify early low-dose long-term colchicine's exact efficacy and safety in AMI patients by more RCTs.

## 2. Methods

### 2.1. Search strategy

RCTs were identified and selected from inception to January 29, 2023. A thorough search was conducted on Web of Science, PubMed, Embase, and Cochrane Library databases without restriction(shown in Supplementary document S1).

### 2.2. Study selection and eligibility criteria

The search strategy used the following search terms: (Myocardial Infarction OR Infarction, Myocardial OR Infarctions, Myocardial OR Myocardial Infarctions OR Cardiovascular Stroke OR Cardiovascular Strokes OR Stroke, Cardiovascular OR Strokes, Cardiovascular OR Myocardial Infarct OR Infarct, Myocardial OR Infarcts, Myocardial OR Myocardial Infarcts OR Heart Attack OR Heart Attacks OR Acute Coronary Syndromes OR Coronary Syndrome, Acute OR Coronary Syndromes, Acute OR Syndrome, Acute Coronary OR Syndromes, Acute Coronary OR STEMI OR ST-Segment Elevation Myocardial Infarction OR ST Elevated Myocardial Infarction OR Non-ST Elevated Myocardial Infarction OR Non-ST-Elevation Myocardial Infarction OR Infarction, Non-ST-Elevation Myocardial OR Infarctions, Non-ST-Elevation Myocardial OR Myocardial Infarction, Non-ST-Elevation OR Myocardial Infarctions, Non-ST-Elevation OR Non-ST Elevation Myocardial Infarction OR Non-ST-Elevation Myocardial Infarctions)) AND (colchicine OR Colchicine, (R)-Isomer OR Colchicine, (+−)-Isomer)) AND (Randomized controlled trial OR randomized OR placebo). Additionally, potential missing RCTs were identified by searching the websites ClinicalTrials.gov and Chictr.org.cn.

Studies were eligible if they assessed the cardiovascular effect of colchicine and compared it with standard treatment or placebo in adult AMI patients (age ≥18 years). Moreover, studies were disqualified if they included (1) animal studies, observational studies, reviews, and meta-analyses, RCTs published only as letters or abstracts, as well as trials of unpublished data; (2) study populations with ACS and stable coronary artery disease; (3) they reported no relevant results.

Two investigators (YFZ and YL) independently filtered the titles and abstracts of the retrieved studies according to the inclusion and exclusion criteria, and subsequently excluded duplicates. Next, the full text of the selected studies was independently filtered.

After excluding duplicate studies, two investigators (YFZ and YL) independently screened the titles and abstracts of the retrieved studies based on the inclusion and exclusion criteria, after eliminating duplicate studies. Next, the full texts of the selected studies were screened independently. Finally, if disagreement between the investigators could not reach a consensus on including a particular survey, a third investigator (RZ) was invited to resolve it. Additionally, We hand-searched the reference lists of the final accepted articles for any related RCTs missed by the search strategy.

### 2.3. Data abstraction

Data abstraction was conducted separately from the selected studies by two researchers (YFZ and YL) and censored by a third researcher (RZ). We contacted the corresponding author via e-mail if additional data were required.

The following data were taken: publication year, first author name, type of RCT, characteristics of the study population, age, sex, sample size, duration and dosage of colchicine, initiation of colchicine, time of follow-up, PCI, antiplatelet, statin, dyslipidemia, diabetes, hypertension, discontinuation of treatment.

### 2.4. Quality assessment and study risk of bias assessment

Two researchers (YFZ and YL) individually assessed quality and bias risk of selected RCTs. RCTs included were assessed for risk of bias with the use of the Cochrane Risk of Bias 2 Tool (22), which includes the following six domains: randomization, participant and personnel blinding, allocation concealment, selective reporting, incomplete outcome data, and other biases. In each RCT, three levels of bias were assessed: low, unclear, and high independently by two authors (YFZ and YL) and scrutinized by a third author (RZ).

### 2.5. Statistical analysis

All analyses were carried out using the Cochrane Review Manager (RevMan 5.4.1; Copenhagen: The Nordic Cochrane Centre, The Cochrane Collaboration, 2020). We calculated the pooled risk ratios (RRs) 、mean differences (MDs), or standard mean differences (SMDs) with 95% confidence intervals (CIs) for dichotomous and continuous data. The mean and standard deviation (SD) were estimated using the methods of Luo et al. and Wan et al. (23) if continuous data were reported as median ± interquartile range (IQR). Heterogeneity in effect size was examined using the *χ*^2^ test and *I*^2^ index. The *χ*^2^ test (*P* < 0.05) or *I*^2^ index of ≥50% indicated significant heterogeneity among the included studies.

Possible publication bias was estimated upon visual inspection of the funnel plots. To assess the possible impact of the data from an individual trial on the overall results, sensitivity analysis was performed using sequential leave-one-out. We computed RRs and 95% CI using a random-effect model when studies had significant heterogeneity; otherwise, a fixed-effect model was selected.

The following variables were analyzed in the subgroup analyses: AMI period (≤3 days vs. >3 days), colchicine dosage (0.5 vs. 1 mg/d), and follow-up duration(<1 year vs. ≥1 year). A two-tailed *P* < 0.05 was deemed statistically significant.

### 2.6. Outcomes

The primary outcome was MACE; the composite outcome included nonfatal myocardial infarction, heart failure (HF), nonfatal stroke, all-cause death, and urgent coronary revascularization. The secondary outcome consisted of the components of the primary outcome; a composite of UA, left ventricular ejection fraction (LVEF), CRP, leukocyte, and neutrophil. Safety and adverse events included gastrointestinal (GI) adverse events and diarrhea.

## 3. Results

### 3.1. Study selection

**Figure 1:**
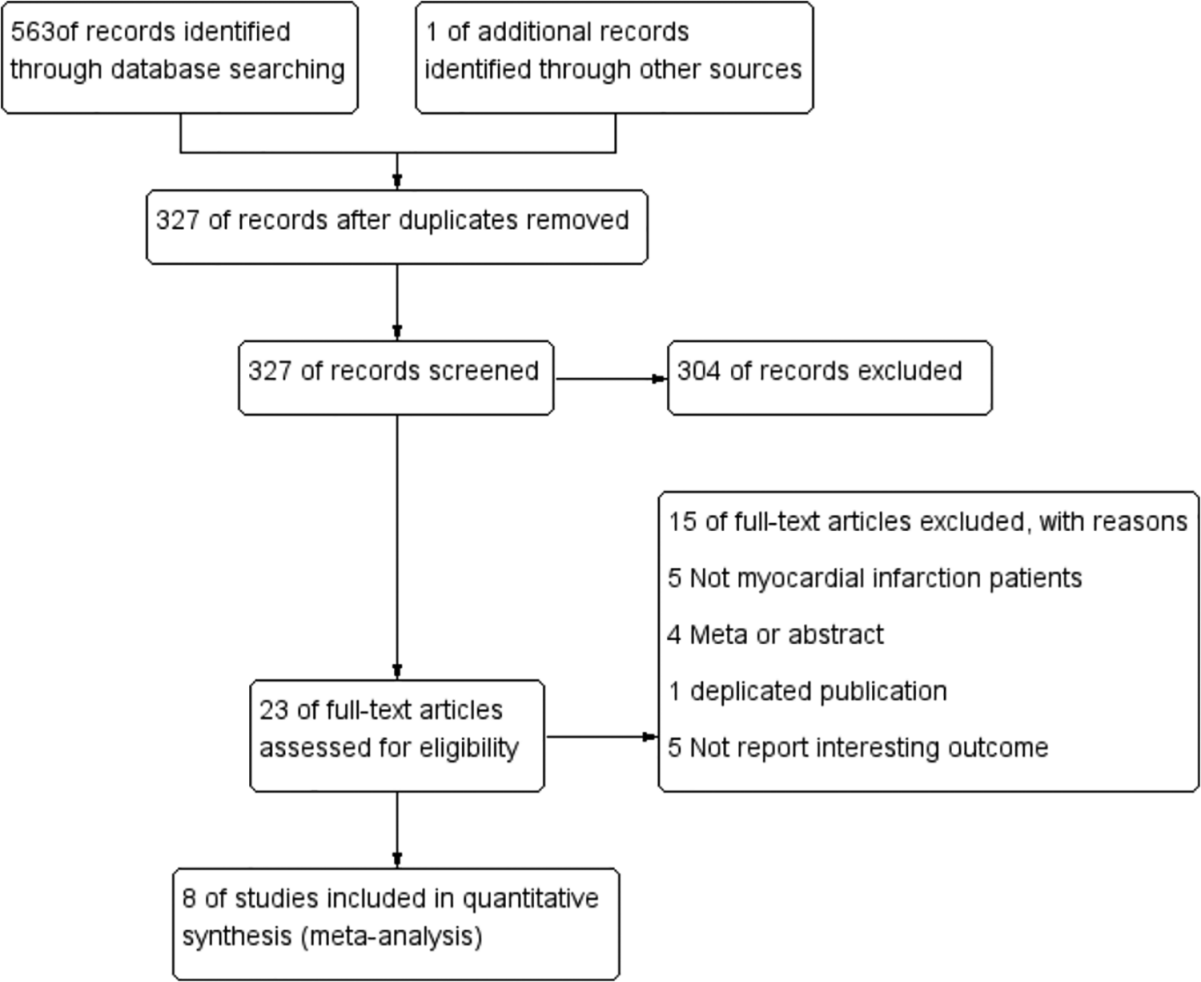
Flowchart of study selection.

Study selection process was shown in Figure 1. The initial search strategy identified 563 potential records. We retrieved and searched for an additional RCT (24) from other sources (15). After eliminating 237 duplicate records, 327 were retained. After reviewing the titles and abstracts of the 327 records, 23 potentially eligible full-text articles were retained. Eight RCTs that met the preset inclusion criteria were eventually selected for analyses, leading to the enrolment of 5,872 patients (colchicine group: control group = 2,938:2,934).

### 3.2. Study characteristics

The key characteristics of the eight RCTs are shown in Table 1. Eight RCTs recruited 80.3% male participants with an average age of 60.42 ± 10.55 years. The prevalence of hypertension was 49.6%, diabetes 21.6%, and dyslipidemia 32.9%. Moreover, 94.5% of patients underwent primary PCI, and 99.6% administered statins and antiplatelet drugs (99.9%). Between the two groups, baseline characteristics varied slightly across the included studies.

**Table 1:** Main characteristics of the included studies.

Reference	Study design	Population	No(T/C)	Male	Age	DM	HTN	Dyslipidaemia	PCI	Stain	Antiplatelet
Akodad et al. (25)	Open label	STEMI patients ≤12 h successfully treated with PCI	44 (23/21)	78%	59.9	14%	43%	36%	100%	NR	100%
Deftereos et al. (26)	Double blind	STEMI patients ≤12 h from the onset of chest pain	151 (77/74)	69%	58	21%	40%	52%	100%	NR	NR
Gholoobi et al. (27)	Double blind	NSTEMI patients ≤12 h	150 (75/75)	52%	61.42	49%	NR	NR	NR	98%	100%
Hennessy et al. (28)	Double blind	a type 1 AMI patients within the prior 7 days	237 (119/118)	77%	61	22%	47%	NR	90%	96%	100%
Hosseini et al. (29)	Double blind	STEMI patients ≤12 h successfully treated with PCI	321 (161/160)	79%	59	0.36	0.4	0.21	100%	NR	NR
Mewton et al. (30)	Double blind	patients with a first episode of STEMI referred for PCI, chest pain ≤12 h	192 (101/91)	80%	61	13%	31%	33%	100%	98%	100%
Tardif et al. (31)	Double blind	MI patients within 30 days completed any planned PRPs	4,745 (2,366/2,379)	81%	60.55	20%	51%	NR	93%	99%	99%
Wasyanto (24)	Open label	AMI patients	32 (16/16)	88%	55.37	22%	47%	13%	NR	100%	NR
Reference	Colchicine	Initiation of colchicine	Duration of administration	Time of follow-up	Intervention(s)	Discontinuation (%)					
Akodad et al. (25)	1 mg QD	within first day of AMI	30 days	30 days	colchicine + conventional treatment vs. conventional treatment	13%					
Deftereos et al. (26)	0.5 mg BD or QD	within 3 days of MI	5 days	5 days	colchicine vs. placebo	15%					
Gholoobi et al. (27)	0.5 mg BD or QD	within 5 days of AMI	30 days	30 days	colchicine + routine medications vs. placebo + routine medications	NR					
Hennessy et al. (28)	0.5 mg QD	within 7 days of AMI	30 days	30 days	colchicine vs. placebo	5%					
Hosseini et al. (29)	1-mg oral loading dose, followed by 0.5 mg QD	the first day of MI	until discharge	1 year	colchicine vs. placebo	8.6%					
Mewton et al. (30)	2-mg oral loading dose, followed by 0.5 mg BD	the first day of MI	5 days	3 months	colchicine vs. placebo	NR					
Tardif et al. (31)	0.5 mg QD	a mean of 13.5 days after MI	19.6 months (median)	22.6 months (median)	colchicine vs. placebo	18.6%					
Wasyanto (24)	0.5 mg QD	patients with AMI	5 days	5 days	colchicine vs. placebo	NR					

MI, myocardial infarction; AMI, acute myocardial infarction; DM, diabetes disease; HTN, hypertension; PCI, percutaneous coronary intervention; PRPs, percutaneous revascularization procedures. QD, quaque die. BD, bis in die. The duration of colchicine administration mostly coincided with the follow-up period; if it did not, it has been indicated in the table. Deftereos, S 2015: 2 mg (1.5 mg initially followed by 0.5 mg 1 h later) and continuing with 0.5 mg BD for 5 days; Patients with <60 kg received 0.5 mg QD for 5 days. Gholoobi, A. 2021: patients <75 kg, or creatinine clearance <50 ml/min: 0.5 mg QD for 30 days and patients >75 kg: 0.5 mg BD for 30 day.

### 3.3. Risk of bias in studies

**Figure 2:**
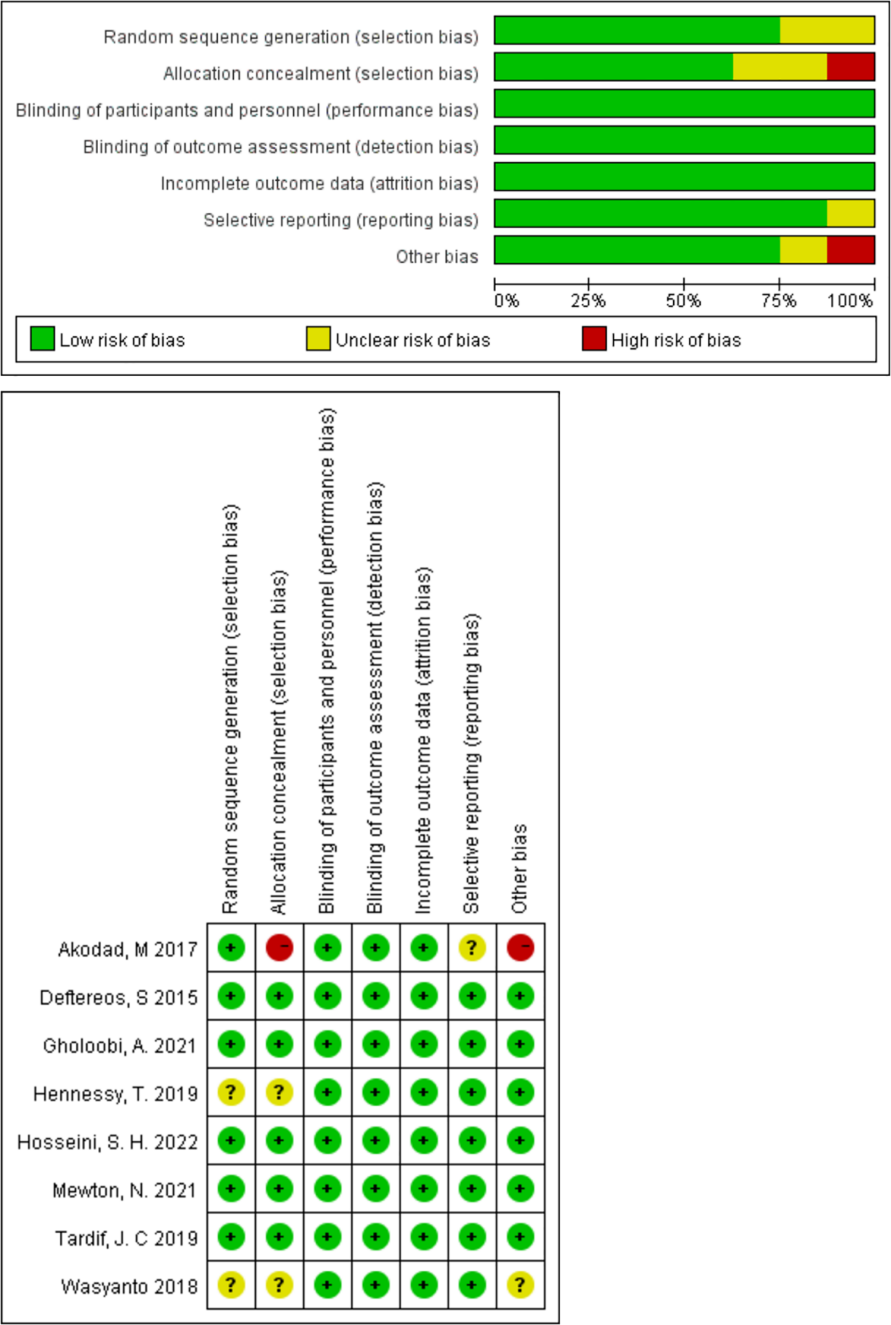
Risks of bias of the included studies.

Individual studies and overall bias summaries are shown in Figure 2. Seven (24, 26–31) of the eight RCTs (24–31) had a low risk of bias. The remaining (25) showed high risk or concern due to insufficient description of the information allocation concealment of patients and baseline imbalance. Funnel plots were constructed for the outcome indicators of concern. Since objective measures as treatment purposes, the selection bias was not considered high risk despite lacking double-blind in the two trials (24, 25).

### 3.4. Results of individual studies

The articles included were published between 2015 and 2022. The colchicine dosage and follow-up varied among the studies. In the colchicine groups, four trials (24, 28, 29, 31) were performed with oral colchicine (0.5 mg) daily, whereas two trials (25, 30) were conducted with 1 mg daily. The remaining two trials (26, 27) used 0.5 or 1 mg daily based on patients' weight. Additionally, two trials (29, 30) received an oral loading dosage of 1 or 2 mg. The length of follow-up varied from five days to 22.7 months.

### 3.5. Primary outcomes

**Figure 3:**
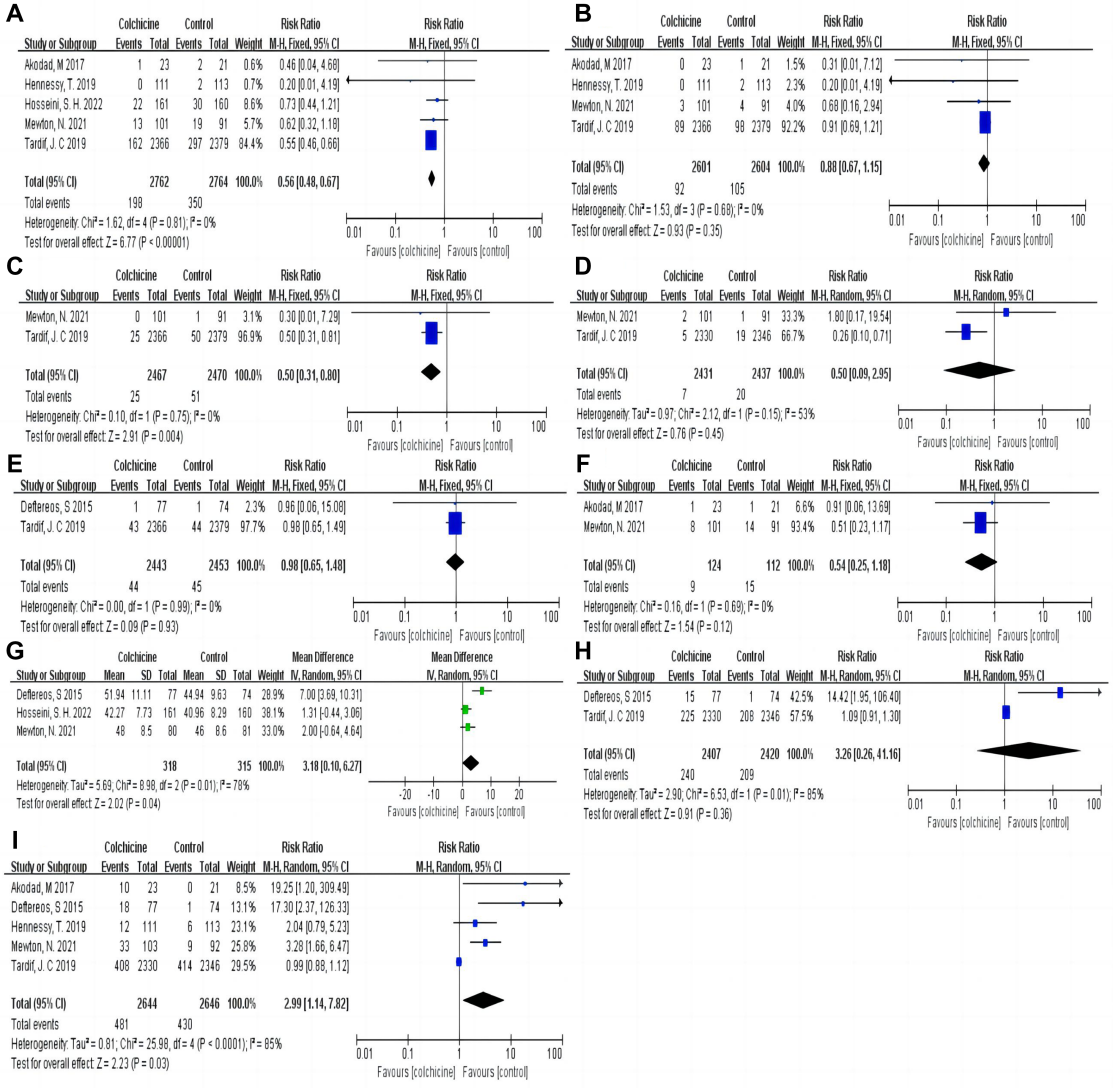
Pooled relative risks and 95% confidence intervals for MACE and the individual components and adverse events in the colchicine and control groups. (**A**) Major adverse cardiovascular events (MACE); (**B**) Myocardial infarction (MI); (**C**) Unstable angina (UA); (**D**) Stroke; (**E**) All-cause death; (**F**) Heart failure; (**G**) Left ventricular ejection fraction (LVEF); (**H**) Diarrhea; (**I**) Gastrointestinal adverse events.

**Figure 4:**
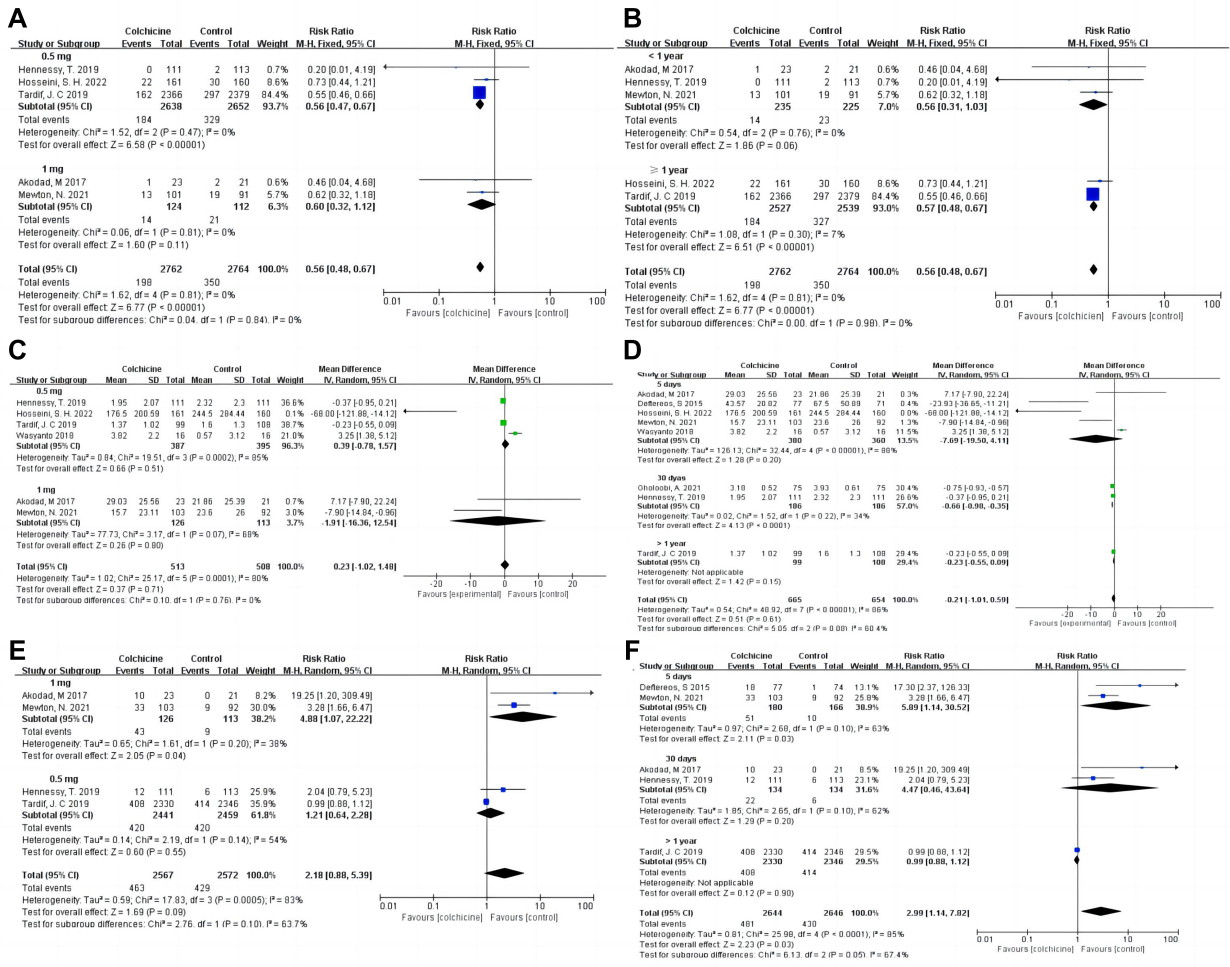
Forest plot showing subgroup analysis in patients with AMI treated with different follow-up duration or dosages of colchicine. (**A**) MACE at different dosages; (**B**) MACE at difference follow-up times; (**C**) CPR at difference dosages; (**D**) CRP at difference follow-up times; (**E**) Gastrointestinal adverse events at difference dosages; (**F**) Gastrointestinal adverse events at difference follow-up times.

**Figure 5:**
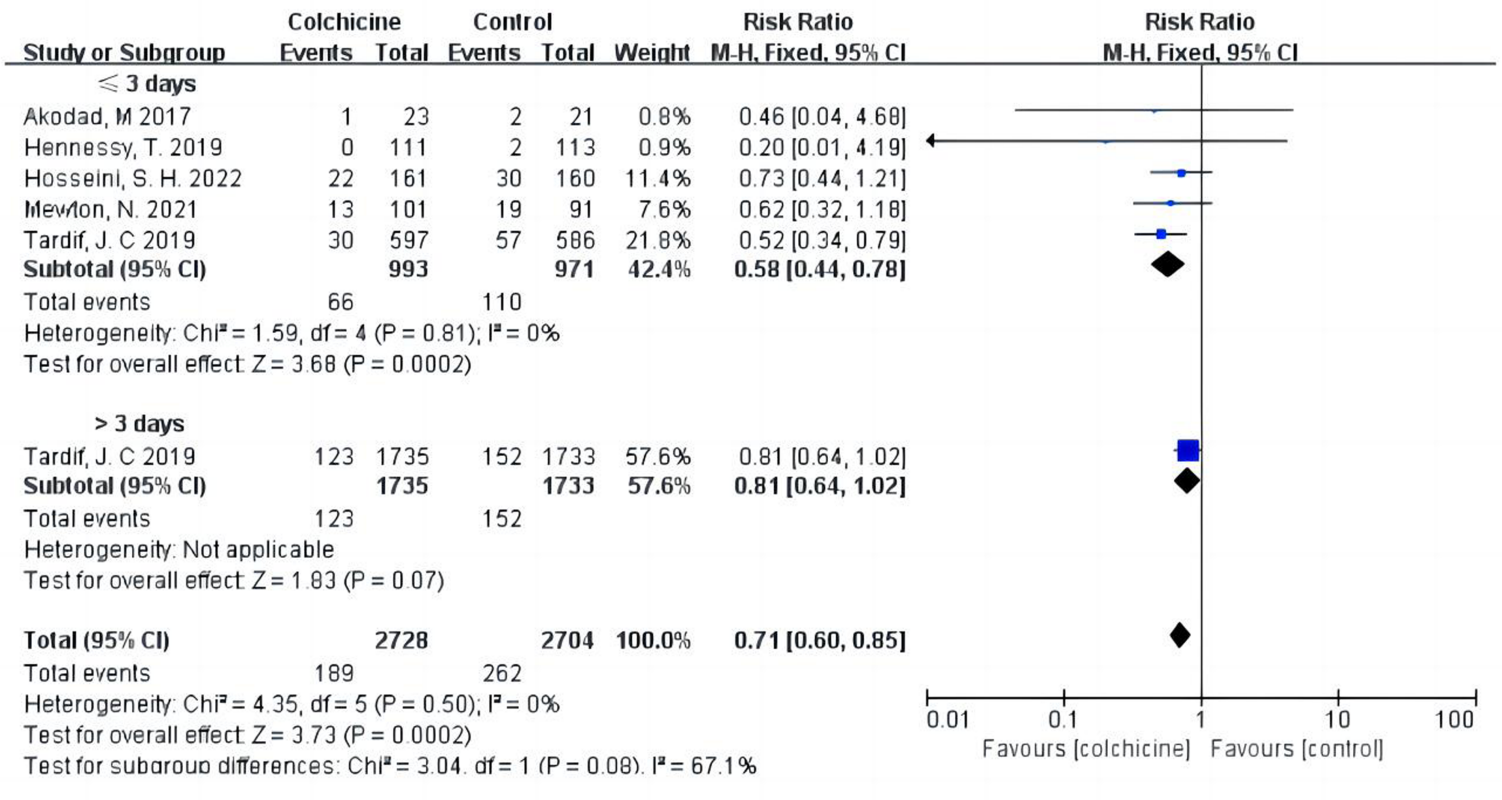
Subgroup analysis for MACE at different periods after AMI.

Our results indicated that colchicine was related to a substantially lower risk of MACE compared to the control group [RR 0.56, (95% CI, 0.48–0.67), *P* < 0.00001; *I*^2^ = 0%; 5 RCTs, *n* = 5,526] in a fix-effects model (shown in Figure 3). We found that colchicine diminished the incidence of MACE (RR 0.57, 95% CI, 0.48–0.67, *P* < 0.00001, *I*^2^ = 7%) when the follow-up period was over one year, but it did not when the follow-up period was less than one year (RR 0.56, 95% CI, 0.31–1.03, *P* = 0.06, *I*^2^ = 0%). Similarly, we noticed that a dosage of 0.5 mg colchicine reduced the incidence of MACE (RR 0.56, 95% CI, 0.47–0.67, *P* < 0.00001, *I*^2^ = 0%), while 1 mg colchicine was ineffective (RR 0.60, 95% CI, 0.32–1.12, *P* = 0.11, *I*^2^ = 0%) (shown in Figure 4). Early administration of low-dose colchicine significantly reduced the risk of MACE within the first three days (RR 0.58, 95% CI, 0.44–0.78, *P* = 0.002, *I*^2^ = 0%) after the incidence of AMI, as compared to that between days 4 and 30 (RR 0.81, 95% CI, 0.64–1.02, *P* = 0.07) (shown in Figure 5).

### 3.6. Secondary outcomes

Compared with the control group, the colchicine group had a lower risk of UA (RR 0.50, 95% CI, 0.31–0.80, *P* = 0.004, *I*^2^ = 0%) and LVEF (RR 3.18, 95% CI, 0.10–6.27, *P* = 0.04, *I*^2^ = 78%). However, there were no significant differences in all-cause death (RR 0.98, 95% CI, 0.65–1.48, *P* = 0.93, *I*^2^ = 0%), MI (RR 0.88, 95% CI, 0.67–1.15, *P* = 0.35, *I*^2^ = 0%), HF (RR 0.54, 95% CI, 0.25–1.18, *P* = 0.12, *I*^2^ = 0%), and stroke (RR 0.50, 95% CI, 0.09–2.95, *P* = 0.45, *I*^2^ = 53%) between the colchicine and control groups (shown in Figure 3).

**Figure 6:**
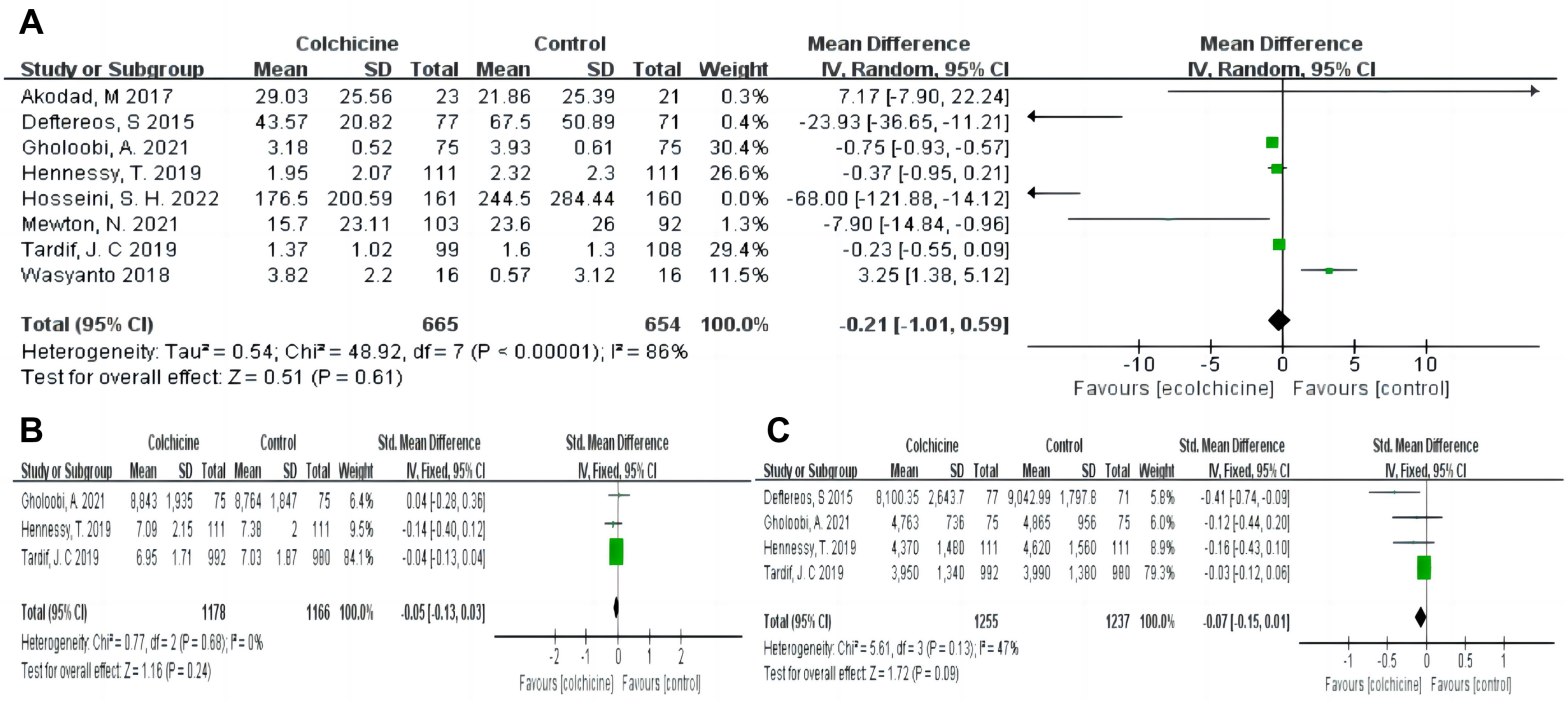
Pooled relative risks and 95% confidence intervals for inflammatory indicators in the colchicine compared with control group. (**A**) CRP; (**B**) Leukocytes; (**C**) Neutrophils.

Moreover, colchicine did not reduce levels of inflammation, such as CRP [MD −0.21, (95% CI, −1.01–0.59), *P* = 0.61; *I*^2^ = 86%; 8 RCTs, *n* = 1,319], leukocytes (SMD −0.05, 95% CI, −0.13–0.03, *P* = 0.24, *I*^2^ = 0%) and neutrophils (SMD −0.07, 95% CI, −0.15–0.01, *P* = 0.09, *I*^2^ = 47%) (shown in Figure 6). No differences in baseline CRP levels were observed. Subgroup analysis revealed colchicine decreased the follow-up levels of CRP by up to 66% when the follow-up period was 30 days [MD −0.66, (95% CI, −0.98– −0.35), *P* < 0.0001; *I*^2^ = 34%]. However, different dosages (0.5 vs. 1 mg/d), and shorter-term or longer-term use of colchicine (5 days or over one year) did not affect CRP levels (shown in Figure 4).

### 3.7. Safety and adverse events

Colchicine was significantly correlated with an increased risk of GI adverse events (RR 2.99, 95% CI, 1.14–7.82, *P* = 0.03; *I*^2^ = 85%, 5 RCTs, *n* = 5,290) in the colchicine group as compared to the control group, with diarrhea being the most common event (RR 3.26, 95% CI, 0.26–41.16, *P* = 0.36, *I*^2^ = 85%) (shown in Figure 3).

The incidence of GI adverse events decreased with an increase in treatment duration. Short-term colchicine use (≤5 days) increased the instances of gastrointestinal adverse events (RR 5.89, 95% CI, 1.14–30.52, *P* = 0.03, *I*^2^ = 63%). Interestingly, there was no difference in the follow-up duration of 30 days (RR 4.47, 95% CI, 0.46–43.64, *P* = 0.20, *I*^2^ = 62%) and over 1 year (RR 0.99, 95% CI, 0.88–1.12, *P* = 0.90) between the colchicine and control groups (shown in Figure 4). Additionally, the risk of GI adverse events increased with increasing doses of colchicine. A colchicine dose of 0.5 mg daily had no significant effect on GI adverse events (RR 1.21, 95% CI, 0.64–2.28, *P* = 0.55, *I*^2^ = 54%)., whereas 1 mg (RR 4.88, 95% CI, 1.07–22.22, *P* = 0.04, *I*^2^ = 38%) caused a 488% increase in RR for GI adverse events (shown in Figure 4).

### 3.8. Sensitivity analyses

**Figure 7:**
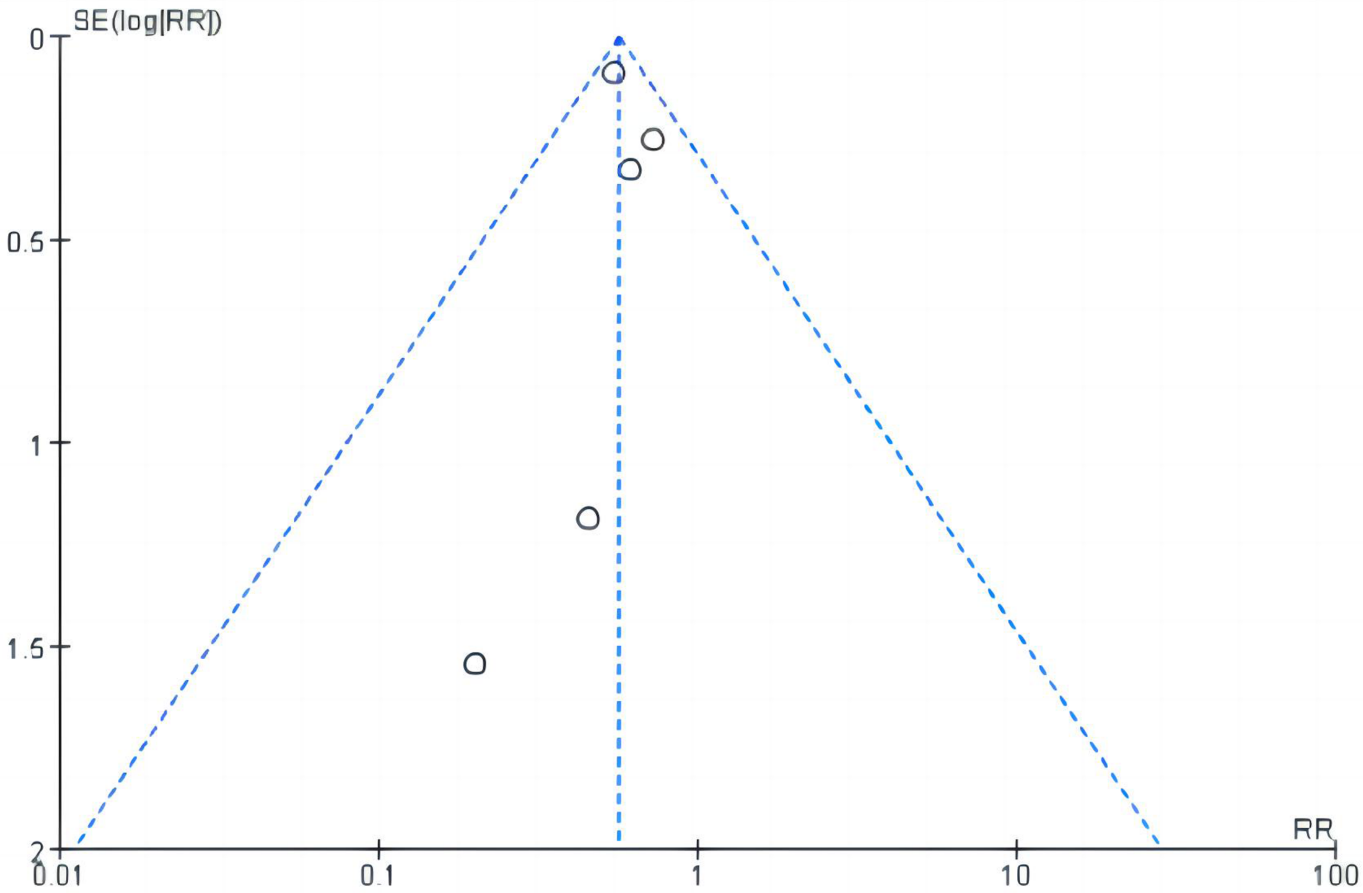
Funnel plot for MACE.

The sensitivity analysis indicated no noticeable change in the overall effect of other outcomes by removing any individual study. In concrete regard, sensitivity analysis did not substantially impact the results, as followings, MACE (RR 0.66, 95% CI, 0.45–0.98, *P* = 0.04; *I*^2^ = 0%), MI (RR 0.46, 95% CI, 0.14–1.52, *P* = 0.21, *I*^2^ = 0%), CRP (MD −0.21, 95% CI, −1.01– 0.59, *P* = 0.61; *I*^2^ = 86%) via eliminating COLCOT (31). Except GI adverse events showed a noticeably higher incidence (RR 4.14, 95% CI, 1.70–10.06, *P* = 0.002; *I*^2^ = 49%) and neutrophils showed lower levels (SMD −0.22, 95% CI, −0.39– −0.55, *P* = 0.01; *I*^2^ = 0%). Our meta-analysis exhibited high heterogeneity and subgroup differences among CRP, LVEF, and GI adverse events (*I*^2^ ≥ 78%). Funnel plot analysis revealed symmetry (shown in Figure 7).

## 4. Discussion

### 4.1. Total effect of colchicine

The present analysis suggested that colchicine in patients with AMI reduced the risk of MACE by 0.56 times, accompanied by 2.99 times higher gastrointestinal risk. Subgroup analysis revealed that colchicine decreased the follow-up levels of CRP (MD −0.66, 95% CI, −0.98– −0.35) and neutrophils (SMD −0.22, 95% CI, −0.39– −0.55) when the follow-up period was 30 days. Compared with other usages, early (≤3 days) long-term (≥1 year) low-dosage (0.5 mg/d) use of colchicine was more effective in reducing the risk of MACE (RR 0.57, 95% CI, 0.48–0.67) without causing more GI adverse events in AMI patients. Cardiovascular events after AMI are still common (32). Colchicine may contribute to changing the clincial status and enhancing the quality of life of AMI patients.

### 4.2. Analysis of the cardiovascular effect

An acute pro-inflammatory response is induced by the ischemia due to myocardial cell injury and death after AMI (33). Following AMI, the co-ordinated effect of activation of the complement cascade and NLRP inflammasomes, production of reactive oxygen species (ROS) et.al. release multiple pro-inflammatory mediators to induce the recruitment of inflammatory cells into the area of infarct, extending the ischemic injury (33).

Sensitive to ischemic and hypoxic injury, mitochondria can sensitively reflect cardiac cell injury and is closely correlated with the severity of myocardial injury in post-AMI (34). Zhang et al. found that oxidatively damaged mitochondria activate large numbers of NLRP3 inflammasomes in rats (34). Toldo et al. discovered that pharmacological inhibition of the NLRP3 inflammasome limits infarct size after AMI in mice, even within 60 min after myocardial ischemia-reperfusion (35).

Persistent and expanded pro-inflammatory response may exacerbate adverse left ventricle remodelling after AMI (33). Koichiro et al. showed that colchicine significantly improved survival, left ventricular end-diastolic diameter and LVEF at 4 weeks after MI in mice via attenuating the expression of pro-inflammatory cytokines and NLRP3 inflammasome, and inhibiting neutrophil and macrophage infiltration (36). Yan et al. (37) showed that activation of the NLRP3 inflammasome led to up-regulation of CRP levels, whereas blockade led to down-regulation. So colchicine may ameliorate inflammation and improve cardiovascular outcomes via the NLRP3/CRP pathway (37). Notably, CRP can predict cardiovascular risk independent of other risk factors (4). This study observed that colchicine rapidly reduced neutrophils and CRP levels at the first month, suggesting a potential association with a later reduction in MACE.

Post-AMI cardiac healing is a complex process, initiated by intense inflammation lasting about 5 to 7 days, followed by resolution and repair with active resolution of inflammation, and finally entering the proliferation phase (4). CRP is a direct acute phase reactant of AMI (38). In post-AMI, neutrophils migrate into the injured myocardium and have a tendency to target the border zone of the infarct, an accumulation that is accentuated by reperfusion. With resolution of inflammation and myocardial repair, neutrophils undergo apoptosis and are subsequently eliminated from the infarct zone (33). We considered the reason why colchicine could not reduce levels of CRP and neutrophils in the first year after AMI can be explained by the fact that the anti-inflammatory effect of colchicine may gradually fail to reach statistical significance with decreasing levels of inflammation in the late-stage cardiac repair. Indeed, late-stage cardiac remodeling results from the incomplete or damaged resolution of myocardial inflammation, accompanied by a greater degree of damage after AMI and amplified over time (4).

Atherosclerosis is a continuous inflammatory disorder within the arterial wall (3, 38). The persistence of lipid accumulation and vascular endothelium injury within coronary arteries and massive cardiomyocyte death, repair of inflammatory cells after AMI lead to ongoing inflammation (3, 4, 38). Disproportionate prolonged, excessive, or inadequate suppression of the inflammatory phase result in persistent tissue damage and improper repair, defective scar formation, increased cell loss, and systolic dysfunction, thereby promoting infarct enlargement, maladaptive remodeling, and ventricular dilation (4). Hence, timely, appropriate, and sufficiently lengthy long-term anti-inflammatory therapy has the potential to improve the prognosis of AMI.

Long-term treatment with colchicine has shown promise in reducing the risk of MACE, primarily by decreasing the incidence of UA and improving LVEF. Colchicine may interfere with neutrophil-platelet interactions for anti-thrombosis (6, 14, 39). Long-term colchicine treatment reduces plaque instability, particularly in low-intensity plaque volumes (6, 28). However, COVERT-MI reported an unexpected three-fold increase in the incidence of left ventricular thrombosis, possibly due to a pro-inflammatory rebound upon early cessation of colchicine therapy, leading to increase left ventricular injury and subsequent thrombosis (30). Additionally, LVEF showed prognostic value in predicting MACEs (40). Colchicine inhibition of post-AMI neutrophils extracellular traps may improve LVEF, attenuated ventricular remodeling, and enhance cardiac function (21, 41).

Our study aligned with a recent meta-analysis that low-dose colchicine decreased the risk of MACE, whereas 1 mg did not (14). Activated neutrophils may transform stable plaques into unstable ones (6). Interestingly, colchicine, highly concentrated in leukocytes, especially neutrophils, binds tubulin to inhibit neutrophil chemotaxis and recruitment (39). Additionally, appropriate changes in inflammation may facilitate cardioprotection (42). Early complete suppression of inflammation may result in the enlargement of the final MI size (42). Therefore, early administration of high-dose colchicine did not reduce myocardial injury and resulted in inflammation compared to the controls. Nevertheless, an excessive early inflammatory response or prolongation is also detrimental to cardiomyocyte repair (42).

Our analysis indicated that the early administration of low-dose colchicine significantly reduced the risk of MACE within the first three days after the incidence of AMI, compared to that between days 4 and 30. A current study observed that colchicine had achieved an 80% reduction in the incidence of MI in CAD settings (16). It suggests that early intervention with colchicine is beneficial for improving cardiovascular outcomes before inflammatory flare-ups cause MI.

The meta-Analyses of Diaz-Arocutipa et al. reported that subgroup analyses of colchicine's dose, follow-up duration, and treatment duration did not show a statistical difference (15). In contrast, our study included more trials (29, 30) with patients with MI occurring within 12 h and completely excluded UA patients. Moreover, the COPS trial included in the previous meta-analysis used colchicine 0.5 mg twice per day and then a dose of 0.5 mg daily for 11 months (21). Early use of high doses may delay the therapeutic time window, resulting in lower-than-expected results.

In the studied population, the rate of all-cause mortality was 1.78%, with cardiovascular deaths accounting for about half of the total causes. Our findings support previous studies on colchicine for coronary artery disease, showing no differences in all-cause mortality and cardiovascular mortality (6, 39).

### 4.3. Analysis of the adverse effect

As expected, the adverse effects of colchicine were mainly due to gastrointestinal symptoms (e.g., diarrhea and nausea), with rare adverse effects such as infection. Patients who cannot tolerate colchicine may discontinue the drug early because of more intense gastrointestinal reactions, even at low daily dosages, leading to higher-than-expected occurrences of MACE. However, 90% of the patients who did not develop early treatment intolerance were administered colchicine for long periods without notable long-term side effects (39, 43), resulting in lower occurrences of MACE. Additionally, long-term colchicine (0.5 mg) administration is difficult beyond safe serum levels despite moderate renal impairment or co-use with most medications. However, oral loading with 1–2 mg of colchicine quickly exceeds safe serum levels and may be fatal (39).

### 4.4. High heterogeneity among CRP, LVEF, and GI adverse events

Our study showed high heterogeneity among CRP, LVEF, and GI adverse events. We conducted analysis to explore the reasons behind this high heterogeneity.

#### 4.4.1. CRP

Colchicine administration for different lengths of time resulted in different anti-inflammatory effects during different stages of post-infarction healing. Due to being at the peak of post-infarction inflammation, the inhibition of inflammation with 5 days of colchicine use did not reach statistical significance. With 30 days of colchicine use, colchicine effectively reduced CRP levels during the resolution and repair stage. The lack of reduction in CRP levels during the first year after AMI can be attributed to the fact that the anti-inflammatory effect of colchicine may gradually fail to reach statistical significance with decreasing levels of inflammation in the late-stage cardiac repair.

#### 4.4.2. LVEF

Proper modulation of inflammation may facilitate cardioprotection (39). Early complete suppression of inflammation may result in an enlargement of the final MI size (39). Studies by Hosseini et al. 2022 and Mewton et al. 2021 were orally overloaded with colchicine. Early administration of high-dose colchicine did not reduce myocardial injury. On the contrary, it might result in inflammation and enlarged final MI size, thereby diminishing the optimal effect of colchicine in boosting LVEF.

#### 4.4.3. GI adverse events

Most participants in studies by Akodad et al. 2017 and Mewton et al. 2021, as well as Deftereos et al. 2015 received a daily oral dose of 1 mg of colchicine. The participants in Akodad et al. 2017 and Deftereos et al. 2015 had more missed visits. It was observed that the participants in Akodad et al. 2017 and Deftereos et al. 2015. On the other hand, patients taking 1 mg of colchicine daily experienced more GI adverse events than those taking 0.5 mg daily. Discontinued the colchicine due to more intense gastrointestinal reactions resulted in lower-than-expected results.

To sum up, combined with the results of the subgroup analysis, we concluded that the high heterogeneity among CRP, LVEF, and gastrointestinal adverse events had few impact on the final conclusion.

### 4.5. Research limitations and prospects

Our meta-analysis has several limitations: (1) The participants of studies existed heterogeneity with regards to the type and severity of diseases (NSTEMI and STEMI), colchicine duration, daily dosage, and loading dose. These factors can lead to misleading conclusions. However, we performed a subgroup analysis consistent with the leading results. (2) Different methodological qualities between open and double-blinded studies likely influence the reliability of results. (3) Since the present study was retrospective, the absence of CRP and neutrophil data in some of the cinical trials may induce bias in the results.

## 5. Conclusion

Our meta-analysis finds that colchicine can decrease the risk of MACE, including UA and LVEF, via anti-inflammation. Furthermore, colchicine is more effective and safe in the clinical setting, especially in early long-term low-dose. However, this meta-analysis still has some limitations. Future studies are desirable to validate our discovery and to provide further insights into the underlying mechanisms.

## Data Availability

The original contributions presented in the study are included in the article/**Supplementary Material**, further inquiries can be directed to the corresponding author.
