# Supplementary material for: Early long-term low-dosage colchicine and major adverse cardiovascular events in patients with acute myocardial infarction: a systematic review and meta-analysis
Source: Front Cardiovasc Med. 2023 Aug 7;10:1194605. doi: 10.3389/fcvm.2023.1194605 (PMC10440701; doi:10.3389/fcvm.2023.1194605)
Supplement: Supplementary file 1 [file Table1.docx]

**Search Strategy**

1. Pubmed:

**Search Results：194** potential records

((((((((((((((((((((((((((((((((((((Myocardial Infarction[MeSH Terms]) OR (Infarction, Myocardial[Title/Abstract])) OR (Infarctions, Myocardial[Title/Abstract])) OR (Myocardial Infarctions[Title/Abstract])) OR (Cardiovascular Stroke[Title/Abstract])) OR (Cardiovascular Strokes[Title/Abstract])) OR (Stroke, Cardiovascular[Title/Abstract])) OR (Strokes, Cardiovascular[Title/Abstract])) OR (Myocardial Infarct[Title/Abstract])) OR (Infarct, Myocardial[Title/Abstract])) OR (Infarcts, Myocardial[Title/Abstract])) OR (Myocardial Infarcts[Title/Abstract])) OR (Heart Attack[Title/Abstract])) OR (Heart Attacks[Title/Abstract])) OR (Acute Coronary Syndromes[Title/Abstract])) OR (Coronary Syndrome, Acute[Title/Abstract])) OR (Coronary Syndromes, Acute[Title/Abstract])) OR (Syndrome, Acute Coronary[Title/Abstract])) OR (Syndromes, Acute Coronary[Title/Abstract])) OR (STEMI[Title/Abstract])) OR (ST Segment Elevation Myocardial Infarction[Title/Abstract])) OR (ST Elevated Myocardial Infarction[Title/Abstract])) OR (NSTEMI[Title/Abstract])) OR (Non ST Elevated Myocardial Infarction[Title/Abstract])) OR (Non-ST-Elevation Myocardial Infarction[Title/Abstract])) OR (Infarction, Non-ST-Elevation Myocardial[Title/Abstract])) OR (Infarctions, Non-ST-Elevation Myocardial[Title/Abstract])) OR (Myocardial Infarction, Non-ST-Elevation[Title/Abstract])) OR (Myocardial Infarctions, Non-ST-Elevation[Title/Abstract])) OR (Non ST Elevation Myocardial Infarction[Title/Abstract])) OR (Non-ST-Elevation Myocardial Infarctions[Title/Abstract])) AND (Colchicine[MeSH Terms])) OR (Colchicine, (R)-Isomer[Title/Abstract])) OR (Colchicine, (+-)-Isomer[Title/Abstract]))

1. Cochrane Library

**Search Results：53** potential records

Search Name:

Date Run: 29/01/2023 12:28:17

Comment:

ID Search Hits

#1 MeSH descriptor: [Myocardial Infarction] explode all trees 11886

#2 (Infarction, Myocardial):ti,ab,kw (Word variations have been searched) 34582

#3 (Infarction, Myocardial):ti,ab,kw OR (Infarctions, Myocardial):ti,ab,kw OR (Myocardial Infarctions):ti,ab,kw OR (Cardiovascular Stroke):ti,ab,kw OR (Cardiovascular Strokes):ti,ab,kw OR (Stroke, Cardiovascular):ti,ab,kw OR (Strokes, Cardiovascular):ti,ab,kw OR (Myocardial Infarct):ti,ab,kw OR (Infarct, Myocardial):ti,ab,kw OR (Infarcts, Myocardial):ti,ab,kw OR (Myocardial Infarcts):ti,ab,kw OR (Heart Attack):ti,ab,kw OR (Heart Attacks):ti,ab,kw OR (Acute Coronary Syndromes):ti,ab,kw OR (Coronary Syndrome, Acute):ti,ab,kw OR (Coronary Syndromes, Acute):ti,ab,kw OR (Syndrome, Acute Coronary):ti,ab,kw OR (Syndromes, Acute Coronary):ti,ab,kw OR (STEMI):ti,ab,kw OR (ST Segment Elevation Myocardial Infarction):ti,ab,kw OR (ST Elevated Myocardial Infarction):ti,ab,kw OR (NSTEMI):ti,ab,kw OR (Non ST Elevated Myocardial Infarction):ti,ab,kw OR (Non-ST-Elevation Myocardial Infarction):ti,ab,kw OR (Infarction, Non-ST-Elevation Myocardial):ti,ab,kw OR (Infarctions, Non-ST-Elevation Myocardial):ti,ab,kw OR (Myocardial Infarction, Non-ST-Elevation):ti,ab,kw OR (Myocardial Infarctions, Non-ST-Elevation):ti,ab,kw OR (Non ST Elevation Myocardial Infarction):ti,ab,kw OR (Non-ST-Elevation Myocardial Infarctions):ti,ab,kw 46663

#4 #1 OR #2 OR #3 46744

#5 MeSH descriptor: [Colchicine] explode all trees 431

#6 MeSH descriptor: [Randomized Controlled Trial] explode all trees 118

#7 (Randomized):ti,ab,kw (Word variations have been searched) 1196555

#8 (Randomized):ti,ab,kw OR (Placebo):ti,ab,kw 1161114

#9 #6 OR #7 OR #8 1289558

#10 #4 AND #5 AND #9 53

1. Embase

**Search Results：125**

**Embase**

**Session Results**

**.......................................................**

**No. Query Results Results Date**

**#6. #5 AND #3 AND #4 125 29 Jan 2023**

**#5. #1 OR #2 349,890 29 Jan 2023**

**#4. 'randomized controlled trial':ti,ab OR 1,095,257 29 Jan 2023**

**'randomized':ti,ab OR 'placebo':ti,ab**

**#3. 'colchicine':ti,ab OR 'colchicine, 22,080 29 Jan 2023**

**(r)-isomer':ti,ab OR 'colchicine,**

**(+-)-isomer':ti,ab**

**#2. 'infarction, myocardial':ti,ab OR 'infarctions, 96,946 29 Jan 2023**

**myocardial':ti,ab OR 'myocardial**

**infarctions':ti,ab OR 'cardiovascular**

**stroke':ti,ab OR 'cardiovascular strokes':ti,ab**

**OR 'stroke, cardiovascular':ti,ab OR 'strokes,**

**cardiovascular':ti,ab OR 'myocardial**

**infarct':ti,ab OR 'infarct, myocardial':ti,ab OR**

**'infarcts, myocardial':ti,ab OR 'myocardial**

**infarcts':ti,ab OR 'heart attack':ti,ab OR 'heart**

**attacks':ti,ab OR 'acute coronary**

**syndromes':ti,ab OR 'coronary syndrome,**

**acute':ti,ab OR 'coronary syndromes, acute':ti,ab**

**OR 'syndrome, acute coronary':ti,ab OR**

**'syndromes, acute coronary':ti,ab OR**

**'stemi':ti,ab OR 'st segment elevation myocardial**

**infarction':ti,ab OR 'st elevated myocardial**

**infarction':ti,ab OR 'nstemi':ti,ab OR 'non st**

**elevated myocardial infarction':ti,ab OR**

**'non-st-elevation myocardial infarction':ti,ab OR**

**'infarction, non-st-elevation myocardial':ti,ab**

**OR 'infarctions, non-st-elevation**

**myocardial':ti,ab OR 'myocardial infarction,**

**non-st-elevation':ti,ab OR 'myocardial**

**infarctions, non-st-elevation':ti,ab OR 'non st**

**elevation myocardial infarction':ti,ab OR**

**'non-st-elevation myocardial infarctions':ti,ab**

**#1. 'myocardial infarction':ti,ab 305,280 29 Jan 2023**

1. Web of Science

**Search Results：191** potential records

[((TS=(Myocardial Infarction OR Infarction, Myocardial OR Infarctions, Myocardial OR Myocardial Infarctions OR Cardiovascular Stroke OR Cardiovascular Strokes OR Stroke, Cardiovascular OR Strokes, Cardiovascular OR Myocardial Infarct OR Infarct, Myocardial OR Infarcts, Myocardial OR Myocardial Infarcts OR Heart Attack OR Heart Attacks OR Acute Coronary Syndromes OR Coronary Syndrome, Acute OR Coronary Syndromes, Acute OR Syndrome, Acute Coronary OR Syndromes, Acute Coronary OR STEMI OR ST Segment Elevation Myocardial Infarction OR ST Elevated Myocardial Infarction OR nastemi OR Non ST Elevated Myocardial Infarction OR Non-ST-Elevation Myocardial Infarction OR Infarction, Non-ST-Elevation Myocardial OR Infarctions, Non-ST-Elevation Myocardial OR Myocardial Infarction, Non-ST-Elevation OR Myocardial Infarctions, Non-ST-Elevation OR Non ST Elevation Myocardial Infarction OR Non-ST-Elevation Myocardial Infarctions)) AND TS=(colchicine OR Colchicine, (R)-Isomer OR Colchicine, (+-)-Isomer)) AND TS=(Randomized controlled trial OR randomized OR placebo) | 191](http://www--webofscience--com--https.webofscience.gzzyy.yuntsg.cn:2222/wos/alldb/summary/d0ad99a3-35bc-4ed2-b34b-020bc4947c71-6ca87caa/relevance/1)
